# Fertility Preservation in Pediatric Age: Future Perspective among Andrological Diseases

**DOI:** 10.3390/life13091934

**Published:** 2023-09-20

**Authors:** Nicola Zampieri

**Affiliations:** 1 Pediatric Surgical Unit, Pediatric Fertility Lab, Department of Engineering for Innovation Medicine, University of Verona, Piazzale Stefani, n. 1, 37100 Verona, Italy; nicola.zampieri@univr.it; 2 Pediatric and Infant Surgery, UniCamillus International Medical University, Via di Sant’Alessandro 8, 00131 Rome, Italy

**Keywords:** pediatric, infertility, andrology, preservation, surgery, innovation

## Abstract

Male infertility is a condition that has always been less studied and known than female infertility. Male infertility is increasingly present and increasingly diagnosed. Although several causes are known, to date about 40% of the causes are considered idiopathic. The worldwide denasality can only be slowed if awareness campaigns are implemented on all the diseases that can alter fertile potential, especially in young adolescents. Male infertility is, in addition, associated with several medical conditions. In particular, the association between infertility and testicular cancer, cardiovascular disease, autoimmune diseases, and genetic diseases is well known. For this reason, fertility preservation should not be proposed or be only oncological in nature, as there are several diagnosable pediatric pathologies that are associated with altered fertile potential to whose patients we could offer a gamete preservation pathway. In this paper we propose our experience on fertility preservation in pediatric andrological diseases.

## 1. Introduction

Infertility is defined as the inability to conceive after one year of regular unprotected intercourse. According to the World Health Organization, 48 million couples globally suffer from infertility, but the numbers may be higher when taking into account low-income countries, where access to fertility services may not be guaranteed. In 50% of cases infertility is attributable to male causes. In 20% of cases it is the sole responsible factor, while in 30–40% it is a co-responsible factor. There is also a reported declining trend in sperm count globally: from 1973 to 2018 the decline was 51.6%. Although the causes of this decline are not yet fully understood, a significant correlation has been found with increasing obesity rates, the Western diet, and exposure to environmental toxins. Male infertility is, in addition, associated with several medical conditions—in particular the association between infertility and testicular cancer, cardiovascular disease, autoimmune diseases, and genetic disorders is well known. Male infertility is a condition that has always been less studied and known than female infertility; the problem of infertility often becomes apparent in adulthood, following tests performed in cases of failure to conceive naturally, when the only viable strategy is through medically-assisted procreation techniques [1,2,3,4,5,6].

The causes of male infertility can be divided into four categories:

- Endocrine and systemic disorders with hypogonadotropic hypogonadism (5–15% of cases)
- Primary testicular defects of spermatogenesis (70–80% of cases)
- Defects in sperm transport (2–5% of cases)
- Idiopathic male infertility (10–20% of cases)

Any disease that can be related to hypogonadotropic hypogonadism, with a deficiency of hypothalamic/pituitary function, can result in a defect in spermatogenesis, failing stimulation with GnRH on the pituitary or gonadotropins on the testis. The consequence of this is the absence of hormonal stimulation on the testis related to spermatogenesis [3,4,5,6,7].

## 2. Spermatogenesis and Andrological Diseases

**Figure 1:**
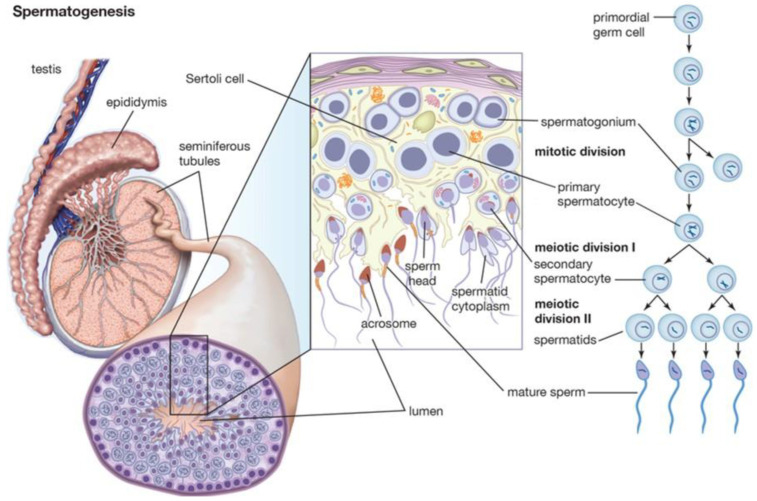
Spermatogenesis and hormonal control.

The term spermatogenesis refers to a set of processes that occur in the seminiferous tubules and result in the production of mature male gametes (Figure 1). The processes are, in temporal order: proliferation of spermatogonia; differentiation of spermatogonia into spermatocytes; meiotic division of spermatocytes into spermatids; release of mature spermatozoa into the tubular lumen. The main cellular actors involved in spermatogenesis are peritubular myoid cells, Leydig cells, Sertoli cells, and germ cells. 

Peritubular myoid cells are cells of mesenchymal origin that provide support to the seminiferous tubules and, due to contractile properties, ensure sperm propulsion through the tubules. Another important function of these cells is the maintenance of the blood–testicular barrier, a structure responsible for separating the basal and adluminal compartments, ensuring immune tolerance in the confrontation of germ cells. Several factors control the activity of these cells, most notably androgen hormones.

Leydig cells are cells of mesenchymal origin, located between blood vessels and seminiferous tubules. Their main function is the production of testosterone, in amounts of about 3–10 mg/day in adults. Intratesticular testosterone levels are on the order of 100 times higher than in blood, and the maintenance of these concentrations is necessary for proper spermatogenesis. Leydig cells are, in addition, the main source of estradiol in humans. The maintenance of a constant ratio of testosterone to estrogen is involved in proper testicular function. The most important factor involved in Leydig cell function is luteinizing hormone (LH). This hormone induces steroidogenesis and inhibits cell apoptosis.

Sertoli cells are supportive cells that drive spermatogenesis. They occupy 20% of the epithelium of the seminiferous tubules and are distinguished from germ cells by their irregular shape. The cytoplasmic offshoots of Sertoli cells support germ cells at different maturational stages. A single Sertoli cell can support about 30–50 germ cells. One of the main products secreted by Sertoli cells is androgen-binding protein (ABP). This protein binds testosterone and dihydrotestosterone with high affinity, ensuring its high concentration in the extracellular space, which is critical for germ cell function. Sertoli cells also act as macrophages, clearing through phagocytosis the extracellular space of spermatid remnants or senescent germ cells. The regulation of Sertoli cell function occurs, primarily, by follicle-stimulating hormone (FSH), androgen hormones, and insulin.

FSH, produced by the pituitary upon stimulation of hypothalamic gonadotropin-releasing hormone (GnRH), activates several intracellular signal transduction pathways that regulate spermatogenesis. Androgen hormones play a role in meiosis and spermatid maturation. Insulin regulates the production of lactate, the main source of energy for germ cells. The latter finding could explain the defects in spermatogenesis that are evident in patients with type I diabetes.

Germ cells are the main actors in spermatogenesis. They are the only human cells that can carry out meiosis. They are distributed in an organized manner in the seminiferous tubules, with the most mature cells occupying the most superficial layers; germ cells are the main actors in spermatogenesis. They are the only human cells that can carry out meiosis. They are distributed in an organized manner in the seminiferous tubules, with the most mature cells occupying the most superficial layers. Primordial germ cells occupy the gonadal crest between 3 to 5 weeks of age, where they differentiate into gonocytes and arrest at the G0 stage of the cell cycle. Several factors, including glial-derived neurotrophic factor (GDNF), fibroblast growth factor (FGF), colony stimulating factor 1 (CSF1), retinoic acid, and DNA methylation, play a role in primordial germ cell differentiation. Between birth and 6 months of age, gonocytes differentiate into spermatogonia. Spermatogonia remains quiescent until puberty, when differentiative processes into spermatozoa begin. Spermatogonia has the dual role of differentiation into spermatozoa by meiosis and self-renewal by mitosis. It is accepted that Sertoli cell-derived factors regulate the fate of spermatogonia toward differentiation, self-renewal, or apoptosis. Primary spermatocytes, produced by mitosis of spermatogonia, move to the adluminal compartment, where they proceed to maturation by DNA recombination. Failure of this stage may explain the cases of aneuploidia that are evidenced by genetic testing in infertile men. After meiosis 1, two secondary spermatocytes are formed, with haploid chromosomal equipment. During meiosis 2, two spermatids are formed from secondary spermatocytes. During spermiogenesis, a series of cytoplasmic and nuclear changes occur, through which spermatozoa are obtained. These changes include acrosome formation, DNA condensation, and tail formation. At the conclusion of the process, the spermatozoon is released into the tubular lumen, at the stage known as spermiation.

Sperm pathology refers to alterations in quality or quantity. Diagnosis is mainly based on seminal fluid analysis. Quantitative alterations affecting male fertility are oligozoospermia (few sperm produced) and azoospermia (no sperm produced). Qualitative alterations potentially affect every portion of the cell, with the most significant from the point of view of fertility being alterations in the tail (impair motility) and head (impair fertilizing capacity).

Identifying normal values for spermiogram parameters is not easy, precisely because individual patients’ values tend to vary widely over several days. In addition, several studies have shown how there are important geographic differences in test results and how even within the same population individual tests can have high variability. These changes are critical, especially for the study and management of many andrological diseases that may alter fertile potential. After understanding all the fundamental passages of spermatogenesis and its regulation, it is possible to understand how various alterations created by external pathologies can affect the correct mechanism. Hormonal alterations will be the cause of the failure of Sertoli and Leydig cells to function, and alterations in temperature and the production of free radicals directly generated by stress on the testis will be the cause of the failure of spermatozoa to mature, and genetic alterations will be associated with more or less complete alterations in molecular regulatory mechanisms [2,3,4,5,6,7].

## 3. Pediatric Fertility Preservation: The Starting Point

From the data reported by the various scientific societies, the incidence of oncological diseases in pediatric age stands at 140 new cases per million aged 0–14 years. In Italy, in the period 2016–2020, about 7100 new cancer diagnoses were reported in children under the age of 14 years and about 4000 new cases were reported in adolescents aged 14–19 years. Overall, despite a rapidly growing epidemiology, the constant refinement of diagnostic methods, efficacy of therapies, and expansion of supportive therapies have resulted in a 5-year survival prognosis for pediatric cancers in excess of 80 percent. As a result, patients surviving cancer diagnosed and treated in childhood (childhood adolescence and young adult cancer survivors—CAYA) represent an increasingly representative proportion of the general population. Starting from the experience in the management of this specific group of patients, a new field of medicine was born, dedicated to providing care in many clinical aspects; in fact, it is reported that nearly 80 percent of these patients report long-term problems of social and clinical impact, with a variable impact on long-term survival and overall mental and physical well-being status.

As described above, testicular health is crucial for future fertility; although testicular cancers are rare in pediatric age, testicular damage in oncology is directly associated with supportive therapies (chemotherapy and radiotherapy) and indirectly to any surgical therapies. The non-reversibility of infertility induced by chemotherapy or radiotherapy and the consequent important impact on long-term quality of life have motivated and pushed research to promote fertility preservation procedures immediately before starting cancer treatments.

It is therefore essential to raise awareness around parents and doctors who care for pediatric patients suffering from cancer regarding the risks of infertility [1,2,3,4,5,6,7,8].

### 3.1. Patients at Risk of Infertility: Oncological View

It is quite clear today that in patients surviving oncology disease, the risk of irreversible gonadal damage changes according to age and at the time of therapy. Radiotherapy and abdominal–pelvic surgical procedures associated with chemotherapy are important factors that alter fertile potential, generating greater risks if combined. For example, we know that not all chemotherapy is toxic to the testicle. Alkylating agents (cyclophosphamide, ifosfamide, procarbazine, busulfane, and analogues) are known to cause damage to gametes, regardless of the phase of cellular proliferation, also destroying spermatic precursors during the first meiotic division. Among surgery, patients who require bilateral gonadectomy or a gonadectomy of a solitary gonad for cancer treatment will be rendered infertile [3,4,5,6].

Various instruments have been proposed to calculate the gonad toxicity risk of combined treatment schemes, including cyclophosphamide equivalent dose (EDC), which consists of a formula that balances and integrates the damage of different alkylating agents normalizing it with respect to the known cyclophosphamide. This instrument therefore allows to estimate the overall toxicity in patients undergoing polychemiotherapy treatment, including several drugs.

For radiotherapy, some important factors must be briefly explained; in patients with abdominal or pelvic or testicular cancer, the gonads may be within the irradiation field of loco-regional therapy; cranial (hypothalamic/pituitary) radiation or central nervous system neoplasms can cause central hypogonadisms, which may impair germ cell function. In males, radiant doses at the testicular level >2 Gy and >6 Gy, respectively, in young adults and children are associated with a significant risk of azoospermia.

### 3.2. Patients at Risk of Infertility: Non-Oncological View

Many treatments such as HSCT (hematopoietic stem cell transplantation) or bone marrow transplantation (BMT) are curative treatment option for patients with SCD (sickle cell disease), thalassemia, bone marrow failure, or other non-malignant conditions. Bone marrow failure syndromes may be present at birth or develop later in life. Some bone marrow failure syndromes are due to an underlying inherited genetic condition, while other bone marrow failure syndromes are due to an acquired cause such as viral or drug/toxin exposures; for example, Fanconi anemia (FA), Dyskeratosis congenita (DC), Shwachman–Diamond syndrome (SDS), Congenital amegakaryocytic thrombocytopenia (CAMT), and Diamond–Blackfan anemia (DBA). HSCT requires conditioning therapy with high-risk chemotherapy and/or total irradiation. Therefore, many patients are at risk for long-term side effects such as impaired pubertal development and impaired fertility, especially for testes disorders. In some cases, previous treatment or the disease itself may have caused spermatogonia loss [7].

Sickle Cell Disease is the most common non-malignant condition for which allogenic HSCT is recommended. It is known that the SCD itself can cause delayed puberty as well as disturbed semen parameters and sperm abnormalities in adulthood. The most common cause for infertility related to SCD is hypogonadism. Fertility problems may also be associated with erectile disorders, sexual problems, testicular ischemia/infarction, zinc deficiency, and small testicular size. SCD patients may have fertility problems related to frequent transfusion (iron accumulation) and for the new therapy based on Hydroxyurea that is an FDA-approved non-alkylating antineoplastic agent that can increase the concentration of fetal hemoglobin and thus prevent the sickling of the red blood cells with the mutated adult hemoglobin. The consequence of this treatment is the worsening of semen parameters due to the reduction of number of spermatogonia in prepubertal patients.

Another non-oncological disease associated with impaired fertility is Beta-Thalassemia; this is the second most common hereditary hematological disorder. Also, for this condition, blood transfusion may alter the iron balance and affect many organs such as the testes and the pituitary gland, causing hypogonadism and testicular tissue damage.

Bone marrow failure is another represented group of benign conditions for which immature testicular tissue is banked. Many of these conditions require HSCT and, therefore, testicular failure [1,2,3,4,5,6,7,8].

Among those patients affect by non-oncological disease, we also have to remember that patients receiving immunosuppression, patients with rheumatological and hematological conditions, and those undergoing solid organ transplantation also have potential infertility when the disease requires gonadotoxic immunosuppressive treatments.

## 4. Pediatric Fertility Preservation: New Diseases and New Aspects 

Although from a clinical social point of view oncological diseases have been the main motivation to talk about fertility preservation in recent years, we can see that in reality there are many other clinical conditions that need to be monitored to rule out future fertility problems. There are still other benign diseases, certainly rarer, that can alter fertile potential, such as chronic granulomatous diseases and rare syndromes such as Kostmann and Wiskott–Aldrich. Many other rare metabolic diseases are at risk of infertility, however, new scenes of discussion open up, especially in relation to the quality of life of patients. This aspect will be discussed later.

Instead, it is crucial to talk about the pathologies that can alter fertile potential in the long term, of which it is essential to start a proper clinical monitoring process. In fact, we know that more than 60% of andrological diseases associated with abnormal fertile potential can be diagnosticated and treated in the pediatric age, or at least a multidisciplinary follow-up could be started (Table 1). Many of these conditions require a multidisciplinary approach, especially as they need to be explained to parents, and the treatment course and likely future repercussions need to be explained.

### 4.1. Cryptorchidism

Cryptorchidism is one of the most frequent diseases treated by pediatric surgeons; the impact of pathology on the fertile potential is real and is amply demonstrated. What is still little known is how much surgical treatment can reduce the risk of impaired fertile potential, especially in relation to the timing of the intervention and the post-operative adjuvant treatment. Cryptorchidism affects about 1–4% of full term and 30% of preterm newborns worldwide. This condition is associated with hormonal defects and increased risk of testicular germ cell tumors.

It has been demonstrated that when the testes are absent in the scrotum during the first years of life, the number of germ cells will also decrease, impairing future fertility.

Many studies have reported that unilateral and bilateral cryptorchidism are associated with abnormal semen analysis and/or azoospermia. Recently, our research group reported that post-operative hormonal treatment improves testicular size and vascularization of the treated testes, suggesting that surgery alone, even if performed in the correct age range, is not enough to improve testicular function.

To decrease the risk of infertility and malignancy, orchidopexy is recommended to permanently anchor the testis into the scrotum. According to the urological and andrological associations, surgical procedure should be performed between 6–18 months, because later orchidopexy has been shown to reduce the testicular volume and the number of germ cells and to increase the risk of infertility. The risk of azoospermia remains high in patients with bilateral cryptorchidism, despite early surgical treatment. To preserve the fertility of these patients while germ cells are still present in their testes, testicular tissue could be harvested for long-term storage during orchidopexy or during a separate procedure [3,4,5,6,7,8,9].

### 4.2. Varicocele

The prevalence of varicocele is greatly increasing, and it is estimated that about 1 in 3 subjects have at least one degree of varicocele during pubertal development, which is a separate procedure. The impact of a varicocele on subfertility is not well understood, because most patients with varicocele are fertile. Varicocele is often associated with alterations in the quality of seminal fluid, with alterations in sperm count, motility, and shape. Even in the age of assisted medical procreation, the treatment of varicocele can improve the chances of fertilization. It is clear that it is not enough to treat varicocele, but patients must be monitored over time by verifying the seminal quality. Although most operated patients have a normal quality of seminal fluid, it is described in the literature that spermatogenesis can be altered and remain abnormal even after surgery. It could be proposed to freeze the seminal fluid to avoid progressive deterioration in the long term.

### 4.3. Genital Disorders

Infections such as epididymitis or orchitis and chronic orchitis are the most common pediatric genitourinary infections that may affect fertility potential. Sexually transmitted infections, such as Chlamydia and Neisseria, have to be considered as important factors for infertility. Long-term sequelae of testicular infection may cause obstructive azoospermia with a secondary infertility with the need to have testicular sperm extraction. Relapsing infections, in addition to generating anatomical damage, alter the testicular parnenchima and can therefore also affect the quality of the contralateral testicle.

Among genital defects, an interesting field is the correlation between genital malformations and infertility; for example, hypospadias and related subfertility is a new association.

The embryologic development of male external genitalia formation is partly dependent on dihydrotestosterone (DHT), which is catalyzed by 5-alpha reductase from testosterone. Under the influence of the sex-determining region of the Y-chromosome, Leydig cell differentiation and the production of Testosterone begin in weeks 9–10. The abnormal function of hormones developed a decreased androgens that have many effects on external genitalia development. For these reasons it has been reported that a higher grade of hypospdias may be associated with abnormal testicular parenchyma with lower androgens and fewer germ cells. Population-based studies have demonstrated a 20% reduction in the likelihood of fatherhood, and increasing severity of the hypospdias was associated with decreasing paternity rates. Hypospadias may be associated with other malformations such as undescended testes and this association may alter the final fertility potential. But there are also other long-term complications, such as penile curvature, erectile problems, and lower sexual desire and sensation, that may be associated with secondary infertility. Also, in these cases, the cryopreservation of sperm may help couples during assisted medical procreation [4,5,6,7].

### 4.4. Testicular Torsion and Microlithiasis

Testicular torsion is the most common urological emergency during pediatric age, especially between 5 and 15 years. The majority of cases occur in the perinatal or pubertal period, and it has been reported that some gene mutation (i.e., RAF1) may predispose boys to neonatal torsion. The mechanism of injury and loss of testes is related to an ischemic event due to blood arrest; proposed pathophysiologies of increasing infertility risk include reperfusion injury by reactive oxidative species after an ischemic event and autoimmunization. There are many studies that report an association between testicular torsion and infertility. There are few long-term studies in the literature on post-torsion testicular quality (the cases in which it was preserved after de-rotation). Although conflicting data exist, it should be remembered that both hormonal alterations, especially of FSH, and seminal fluid alterations may be related. This is because a damaged testis (reduced volume and presence of microcalcifications) may also alter the function of the contralateral testis. In our clinical experience, seminal fluid results better in monorchid patients than in patients with de-rotated but morphologically altered testis.

In this setting, therefore, it is critical to understand what can be done to preserve fertile potential, suggesting the cryopreservation of seminal fluid or testicular tissue harvested during surgery. We need to add more information during surgery regarding if it is better to leave the de-rotated testes or to remove it.

Testicular microlithiasis (MT) usually represents an occasional ultrasonographic finding that shows a typical appearance with multiple, tiny, bright hyperechoic spots of the testicular parenchyma, diffused or localized, usually without posterior acoustic shade, involving one or both testicles. Based on the number of hyperechogenic foci per ultrasound field of view, three main forms of MT can be identified: limited form if the foci are <5, classical form if >5, Florida form if countless. An ultrasound can also be described as a “snowstorm” or “starry sky”. Although the MT is considered to be a substantially benign finding (a correlation has also been found with non-neoplastic pathologies, such as cryptorchidism, epididymitis, varicocele, and testicular torsion), as suggested by some authors it should be considered a PRE malignant condition, given the frequent association with in situ testicular carcinomas and testicular germ cell tumors, in particular seminomas. With a frequency of 45 cases per 100 males suffering from testicular cancer, seminoma is the most common testicular neoplasm. The correlation between malignant tumors and MT is variable and controversial and appears to occur more frequently in the infertile male population. MT and infertility could share pathogenetic mechanisms related to testicular dysgenesis syndrome. However, to what extent the presence of MT in infertile men effectively confers a significantly higher risk of testicular cancer remains to be ascertained. In these patients it could be suggested to preserve the sperm rather than the testicular tissue (to avoid eventual malignant cell transplantation).

### 4.5. Genetic Factors and Male Infertility

Male fertility is a multifactorial condition that depends on the interplay between several genetic factors, epigenetic factors, transcriptional regulation, and environmental factors. As we have seen, many pre-existing clinical conditions can also alter the hormonal balance, consequently altering fertility. Altered molecular production and transcriptional alterations can permanently or progressively alter spermatogenesis and consequently generate infertility during adulthood. The clinical manifestation of these genetic alterations is evidenced by altered embryological development, altered genitalia, altered germ cells, altered sperm development, and structural alterations of gametes. Some authors have reported that genetic factors may be involved in and responsible for nearly 30% of male infertility. In the context of infertility, about 10% of patients have alterations in the karyotype; among these conditions the most common syndromes are Klinefelter syndrome and congenital adrenal hyperplasia.

Klinefelter syndrome (KS) has an incidence of 1–2/1000 newborn males and it is the most frequent sex chromosome abnormality in the human. The phenotype of KS is highly variable; KS patients are tall with gynecomastia, neurocognitive, and psychosocial problems, cryptorchidism, small testes, and hypogonadism. This syndrome is also characterized by a progressive loss of germ cells during pubertal development with secondary testicular fibrosis; progressive degeneration of seminiferous tubules and hyperplasia of Leydig cells have also been reported. Over 90% of patients with KS are diagnosed during adulthood with the presence of azoospermia. Although prenatal diagnosis may increase the number of early diagnoses, to date, compared to the number of patients registered in the clinical archives of the syndrome, there must still be many adults who do not know they are suffering from the syndrome [7,8,9].

Many studies in the literature have reported that patients with KS can have active spermatogenesis up to an age between 20 and 25 years; about 30% of patients with KS, and azoospermia, have the possibility of finding sperm at the testicular biopsy during assisted medical procreation procedures, and these data are important to report to patients. However, with advancing age it seems less likely to obtain sperm from biopsy. It is therefore crucial for these patients and for parents to offer clear and safe management. Starting from a prenatal diagnosis, with the correct counseling, we can follow patients with the best follow-up, proposing both the cryopreservation of testicular tissue and proposing the cryopreservation of sperm before the definitive alterations occur.

Congenital adrenal hyperplasia is caused by enzymatic deficiencies in the adrenal steroidogenesis pathway that leads to impaired cortisol biosynthesis. There are several clinical presentations, with the most frequent being 21-hydroxylase deficiency. High adrenal androgens concentration in affected males can lead to hypogonadotropic hypogonadism. Precocious puberty and gonadal disfunction are the most frequent clinical symptoms. More than 35% of patients have oligospermia or azoospermia and a higher risk of testicular adrenal rest tumors is present in more than 35–40% of patients. The general prevalence of adrenal rest in males with adrenogenital syndrome is 37% but, as just reported, with a higher prevalence in more severe forms of enzyme deficiency. In fact, 80% of patients with adrenal rest have a severe form of adrenogenital syndrome and only 20% have a mild form of disease. Usually these are lesions already present in children who tend to increase in number and size during puberty.

Adrenal rest is a benign, intratesticular tumor with functional and histological characteristics similar to adrenocortical cells. They probably stem from a population of pluripotent adrenal-like (adrenal-like) stem cells derived from the urogenital crest, which are already present in the testes during embryonic development. These cells are fetal precursors of Leydig cells that express receptors (for ACTH, LH, and angiotensin II) and can counteract adrenal proliferation and differentiation when subjected to high levels of ACTH, as happens precisely in the condition of congenital adrenal hyperplasia.

Undoubtedly, ACTH plays a crucial role: exposure to high ACTH levels is necessary for the development of adrenal rest tumors. But this exposure must be premature (probably already in prenatal) and chronic. In fact, in patients where high levels of ACTH appear in adulthood, adrenal rest is not found. Further evidence of the importance of ACTH in this condition is given by the fact that adequate treatment of adrenogenital syndrome, which usually reduces the level of ACTH, can also involve dimensional regression of adrenal rest.

It is not excluded, however, that other molecules may also play a role in the proliferation of these embellished cells. For example, LH, with its peak in pubertal age, could explain the increased incidence of testicular adrenal rest tumors in this particular age group.

The diagnosis of adrenogenital syndrome usually precedes the clinical finding of testicular adrenal rest. As mentioned, in fact, these are benign tumors, often bilateral (in 77% of cases) but, especially in the initial stages, are not palpable and are completely asymptomatic, so they can escape clinical palpation. However, these are the main causes of infertility in male patients suffering from congenital adrenal hyperplasia, as they can compromise sperm production over time.

Reduced fertility depends on many factors. First, adrenal rest determines a progressive obstruction of seminiferous tubules, resulting in lymphocytic infiltration and peritubular fibrosis and subsequent and irreversible testicular damage with obstructive azoospermia and infertility, as well as possible testicular pain resulting from prolonged compression of healthy testicular tissue. Reduced fertility can also be caused by androgen production by adrenal rest. Such hyperproduction of adrenal androgens can interfere with testicular function both locally and centrally, resulting in hypogonadotropic hypogonadism.

Surgical treatment (testis-sparing surgery) does not result in significant post-surgical improvement of testicular function, nor does it rule out a possible reappearance of adrenal rest in the future. Therefore, such treatment is indicated only in the case of failure of medical therapy or severe painful symptoms at the testicular level. Adolescent males with this problem, who often have poor therapeutic compliance, should always be told the risk of developing adrenal rest and especially their potential impact on future fertility; the possibility of cryopreservation of seminal fluid should be explained. An annual early (8 years old) and biennial ultrasound screening until adulthood is still recommended. Essentially, there is currently no specific therapy that can treat or prevent the formation of adrenal rest, but it is essential to monitor these patients.

### 4.6. New Aspects: Patients with Gender and Sex Diversity

Disorders and differences of sex development (DSD) and transgender patients with gender dysphoria are old but bring a new future perspective of fertility preservation. Differences of sex development occur when there is incongruence among the chromosomal, gonadal, or phenotypic sex of an individual. At present it is better to define them as “differences” rather than “disorders”.

The main factors affecting the fertility of these patients are:

1. Abnormal gonadal development;
2. Gonadectomy performed for the risk of malignancy;
3. Abnormal hormone production;
4. Potential discordance between gonadal type and gender identity.

Transgender subjects include those who identify with a gender other than the birth-assigned sex. Gender dysphoria is the diagnosis used to describe psychological distress associated with being transgender, and medical treatment of these patients may alter their future fertility. Many transgender adolescents that initiate hormonal treatment may have germ cell maturation failure. Therefore, fertility preservation options should be offered prior to starting any treatment.

## 5. Pediatric Fertility Preservation: Which Method? 

Regardless of pathology and the need to preserve fertile potential, there are two main methods in the male to preserve gametes: cryopreserve seminal fluid or cryopreserve testicular tissue. Cryopreservation of sperm can start with direct collection or through testicular biopsy and direct extraction (Testicular sperm extraction—TESE). Among these two methods some important distinctions should be made, especially in the case of pre- or post-pubertal patients. As we have seen previously, depending on the pathology to be treated, different methods could be used and proposed, especially considering the needs of patients [1,2,3,4,5,6,7,8,9,10,11].

**Figure 2:**
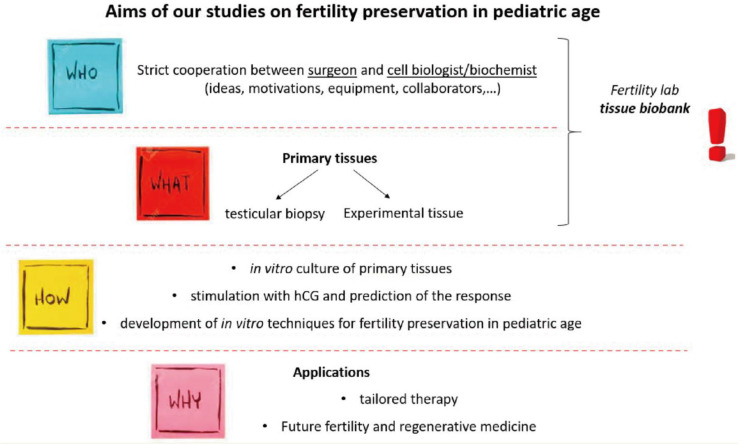
Fertility preservation methods.

**Figure 3:**
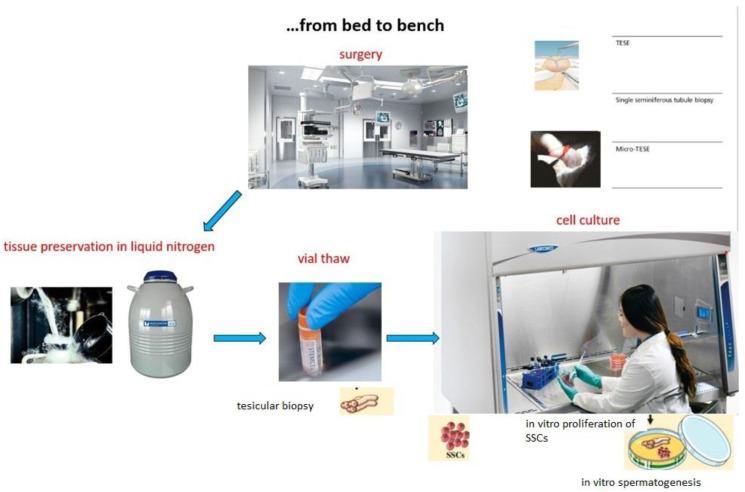
Fertility preservation: the strategy.

In pre-pubertal or post-pubertal adolescents, male fertility preservation can be achieved through cryopreservation of an ejaculated semen sample. When this is not possible due to the patient’s age, anatomical problems, in case of psychological difficulties, or religious reasons, sperm collection can be carried out with a biopsy (Figure 2 and Figure 3). For example, it has been estimated that about 50% of patients with cancer, before beginning therapy, have sperm in the testicular tissue; the obtained sperm can be frozen and used in the future for assisted medical reproduction procedures. Fertility preservation in the pre-pubescent patient is to date experimental. As these patients have not yet started pubertal development, they do not yet produce sperm and have no mature gametes within the testicular tissue. However, the cryopreservation of testicular tissue, using standard freezing techniques, may be proposed for future use. But new approaches are reported in the literature and since the 1990s, several studies have proposed the use of spermatogonial stem cells as a way to study to preserve fertility in young patients; some experiments have shown that the transplantation of these new cells activates spermatogenesis, ensuring the production of the germ line [6,7,8,9].

### 5.1. Testicular Tissue Cryopreservation

There are several aspects to deal with, especially regarding the surgical procedure. It should be explained to patients and families that the testicular biopsy procedure can have post-operative complications, including hematoma and infection. These procedures, especially in the pre-pubescent patient, are performed with anesthesia or mild sedation.

There are several studies in the literature demonstrating the complete safety of the procedure without problems with long-term function and hormonal set-up, especially of the testis. Generally, this procedure is performed in only one testis, however in special cases it can be performed on both. Considering the possible procedure during other surgeries, as we will explain during the discussion, a second surgery may not be necessary. It is therefore crucial to properly select the subjects who need biopsy, especially by arranging the timing for proper management; especially after biopsy, patients should be monitored over time, including during pubertal development, for early dysfunction such as altered parenchyma or increased FSH related to testicular fibrosis.

### 5.2. Future Innovation

Although fertility preservation programs in the pre-pubertal patient are active in several research centers worldwide, there are still important walls to overcome, such as the possibility of restoring spermatogenesis with cryopreserved tissue.

One potential approach for fertility preservation could be to protect germ cells and structural cells of the testis during the administration of therapy for cancer pathology.

This approach, which is termed “ferto protection”, has great potential but is little studied; the theory is to generate hormonal suppression to preserve cell maturation, however, to date, few studies exist in the literature with poor results.

An interesting approach, on the other hand, is to restore spermatogenesis; many techniques to restore spermatogenesis have been proposed, and most require germ cells or testicular tissue to be directly harvested prior to treatment by a testis biopsy before gonadotoxic therapy.

One of the proposed techniques is the testicular tissue culture. For the correct spermatogenesis, the associated activity of germ cells and interstitial cells and testicular microenvironments is fundamental. The aim is to achieve a maturation of germ cells without the need for support of seminiferous tubules. However, this procedure may require external support for the administration of hormones necessary for the progression of cell differentiation.

#### 5.2.1. Testicular Tissue Grafting and Xenografting

Testicular tissue Transplantation has not yet emerged as a therapeutic option; although several animal studies have shown that it is possible to re-establish spermatogenesis after testicular tissue is grafted, there are no studies on humans.

The transplantation of cryopreserved testicular tissue has obtained greater results if transplanted into the scrotum or directly into the residual testicle; these data may confirm the role of testicular disease affecting testicular temperature and consequent function. The possibility of having good parenchyma if transplanted into the scrotum can also be explained by the activity carried out by gubernaculum and peritesticular cells (we will discuss this in the next paragraphs).

#### 5.2.2. Spermatogonial Stem Cell Transplantation and Pluripotent Stem Cell—The Role of In Vitro Differentiation of Male Germ Cells

In the last century, since the 1990s, several studies have been published on spermatogonial stem cell transplantation. These scientists removed the testicles of the animals and created a suspension containing spermatogonial stem cells that was then injected into the seminiferous tubules of the infertile receiving testicle. After several days, the presence of a few steps of spermatogenesis was shown. This technique ensures the resumption of spermatogenesis. The use of this technique is certainly promising, especially because they are present at all ages and can be transplanted easily [5,6].

Stem cells are undifferentiated or partially differentiated cells that have the potential either to differentiate into other cells types or to proliferate as the same stem cell type.

Pluripotent stem cells are cells that have the ability to self-renew or transform into cells of the three embryonal leaflets. Induced pluripotent stem cells are types of cells that are created directly from somatic cells. This technology, reported by Yamaka in 2006, has brought a fundamental scientific innovation. Much subsequent research has shown the possibility of differentiating these pluripotent cells to create germ cells. These studies are still conducted on animals, but in the medical literature long-term results are already present, with the formation of stable and live offspring born. Some offspring developed neck cancer or early death, confirming that the procedure still needs to be structured [5,6].

Again, in vitro propagation of SSCs (Spermatogonial stem cells) followed by auto-transplantation into the seminiferous tubules via rete testis is considered the only method for the restoration of fertility with the potential to have natural conception. After transplantation, the SSCs will colonize the testis, undergo spermatogenesis, and provide continuous sperm production, allowing natural conception to occur.

Because in pre-pubescent patients the number of SSCs is reduced, the multiplication and propagation of these cells will be necessary for self-transplantation. Some studies have shown the efficacy of this technique in humans; SSCs of adults and pre-pubescent patients have been put uncultivated in vitro and then transplanted on experimental testicle generating proliferation of the same. Through these studies, however, other cells such as testicular structures proliferated, so they could not be used.

The future of procreation is therefore not only restoration of spermatogenesis but also in vitro differentiation of the primary spermatocytes in haploid cells. From these cells, different lines of spermatogenesis can develop.

The maturation of the cells of the germinal line can take place in vitro but could also take place in structures similar to the testicles and organoids, which are capable of physiologically supporting the development of sperm. Although the use of spermatids in humans is demonstrated in the literature, the use of a more differentiated cell should be preferred. At present, the mouse is the only species in which complete spermatogenesis has been obtained.

## 6. Ethical Consideration

In the pediatric patient, proposing fertility preservation depends on the patient’s age, pathology, and life expectancy. Generally, and especially in the pre-pubescent patient, consent should be sought from the parents, although some adolescents may already understand the importance of the procedures and the long-term goal and management. From an ethical point of view, the procedure must be explained with the patient’s degree of understanding; in the case of non-oncology patients, for whom the motivation currently seems to be disconnected from the survival and therapeutic harm of chemotherapy, it is crucial to introduce how different andrological diseases can alter fertility potential. In an oncology setting, the most important ethical aspect is to define how the choice to perform fertility preservation influences the initiation of cancer therapy. There are therefore aspects for and against preservation from an ethical point of view, for example, we can list that fertility preservation may be the only chance to preserve the biological possibility of becoming a parent and the possibility of avoiding other hormone therapy to try to stimulate spermatogenesis. This may reduce future stress regarding the idea of being infertile [12,13].

Contrary to these aspects, the procedure could be considered unnecessary, and the surgical procedure could be associated with clinical complications and thus delay the initiation of therapy. With respect to the adolescent patient, the parents’ decision may not coincide with the patient’s choice, and one of the major questions is what to do and how to handle the frozen tissue or frozen semen of patients who do not survive the disease.

These procedures, applied to patients with alterations of gender and sex differentiation or assignment of discordant sex at birth, could generate many social and ethical debates on the need to offer such procedures or whether they really should be proposed [12,13].

One of the most interesting and practical techniques, therefore, could be the use of gametes derived from stem cells, which with the new techniques could also be generated from non-testicular tissue, thus making the surgical procedure unnecessary.

## 7. Financial Consideration and Prospective Management

From a strictly financial point of view, all these procedures, whether experimental or already clinically used, have costs that must be borne by families or by the national health system. As for adults, all procedures for the preservation of testicular tissue or gametes require storage inside laboratories, whether private or public. It is therefore clear that the costs of management are different from state to state, from city to city, and from research centers to other research centers. Costs may also influence a parent’s decision to continue with the procedure and thus to suspend the whole route. It is not possible today to know how much the procedure can cost the individual patient, especially because he may never use frozen gametes or frozen testicular tissue. There are no studies in the literature that have used frozen tissue or gametes in pre-puberty patients.

There are very few states and research centers that carry out the activity of preserving fertility in children. Many studies report that the first survey of research centers that proposed testicular tissue cryopreservation is that of Snape et al., 2015. At the time of the survey in the centers of the study, 260 samples were from pre-pubescent patients, while in 2019 the samples increased to over 1000 patients. This increase can be secondary both to the progressive increase of the procedures performed not only for oncological pathology but also for other pathologies, for the constant adherence to the project of preservation of fertility [8,9,10,11].

Currently, worldwide, the age and range of patients treated varies from a few months to 18 years; it is essential to understand that globally it is necessary to standardize the lines of research and the type of patients affected in order to improve the long-term approach.

## 8. Our Experience and Future Perspective

Recently, the results of scientific research on pediatric andrology have taken continuous research on the preservation of fertility to another level. Such a procedure is not only related to oncology, but there are several pathologies that may require this approach. For over 15 years our research group has proposed the study of pediatric andrological diseases from a new point of view, researching the mechanisms that are associated with the altered fertile potential and preventing long-term damage.

We have studied varicocele in all molecular and biological aspects, and still many studies are necessary. However, even today, despite surgery, some patients have alterations in the quality of seminal fluid and require medically-assisted procreation as adults [13,14,15,16].

Testicular diseases are perhaps the most fascinating to study, namely cryptorchidism and torsion of the testicle. From a general point of view we have seen how cryptorchidism is associated with impaired fertile potential and that testicular quality is related to the age of the intervention. Again, we have shown how early intervention and the use of adjuvant therapies can improve testicular quality and function.

In the lab, we demonstrated how many testicular pathologies affect both testicular vascularity and hormone control. In these patients, offering both preservation of testicular tissue and a clinical path until the control of seminal fluid is crucial to exclude future fertility problems [14,15,16].

For the torsion of the testicle, we have shown that this pathology is not only related to the acute event but is a constant problem until adulthood. Qualitatively, the seminal fluid of patients with torsion of the testicle and with testicle that remained in place is worse, with many values altered. Furthermore, in vitro, we showed that, using chorionic gonadotrophin, we can improve trophism and testicular vascularization.

Our research groups (Pediatric fertility lab and Ilaria Dando lab), through the beginning of three important clinical studies approved by the ethics committee (ANDRO-PRO—4206CESC, PRP 3.0—Prog. 4159CESC, FePo 2.0—Prog. 3072CESC), began the storage of testicular tissue and peripheral blood samples of pediatric patients with testicular disease and/or cancers. Since January 2022, 101 testicular tissues have been cryopreserved, including three samples for cancer disease in pre-pubescent patients. Most patients suffered from cryptorchidism, testicular torsion, varicocele, and DSD (differences of sex development) [14,15,16,17].

## 9. Discussion and Final Considerations

Many andrological diseases diagnosed in pediatric age are responsible for more than 60% of the infertility of the adult male. We have the possibility to preserve fertility at different times of life with minimally invasive methods, although this requires surgical procedures. Many pathologies are still treated surgically, so proper pre-operative counselling could avoid further procedures. The multidisciplinary approach is crucial for the proper management of these patients, and from a clinical point of view, the surgeon can propose the procedures with relative urgency. It is essential that oncologists should speak and explain to parents as soon as possible about the possibility of preservation to avoid unnecessary delays and maybe begin preservation when the first chemotherapy treatment has been performed. This information should be in the public domain, and the importance of these methods should be platformed on social media. Many diseases are already known and are already widely associated with infertility; however, there is still discussion regarding the need to understand the lesser-known pathologies that are still associated with infertility. Proposing performing cryopreservation of the tissue to the patient who is going to be subjected to orchidopexy could be the first step to better manage this pathology. From a strictly scientific point of view, more research has to be conducted to develop new methods for cryopreservation and to “create” the cells of the germinal line.

Through the continuous study of different diseases, and clinical prevention, it will be possible in the future to guarantee fertility to patients suffering from pathologies that today seem to be unconditionally associated with infertility.

## Figures and Tables

**Table 1:** Diseases and fertility potential: description of diseases and medical or surgical treatment.

Andrological Diseases	Treatment	Fertility Potential Storage
Varicocele	surgery	Semen preservation Testicular biopsy
Undescended testes	surgery	Testicular tissue biopsy Spermatogonial stem cells Germ cell maturation
Testicular torsion	surgery	Testicular tissue biopsy Semen preservation
Klinefelter and genetic disorders	Medical treatment	Testicular tissue biopsy Semen preservation Spermatogonial stem cells
DSD Disorders/differences of sex development	Surgery and medical treatment	Testicular tissue biopsy Spermatogonial stem cells Germ cell maturation
Oncological diseases Hematological diseases	Surgery and medical treatment	Testicular tissue biopsy Spermatogonial stem cells Germ cell maturation
Gender dysphoria	Surgery and medical treatment	Cryopreservation of semen Testicular tissue biopsy

## Data Availability

The data can be shared upon request.
